# Effect of electrical stimulation of central nucleus of the amygdala on morphine conditioned place preference in male rats

**DOI:** 10.22038/IJBMS.2022.62133.13751

**Published:** 2022-05

**Authors:** Zahra Jokara, Saeed Khatamsaz, HojjatAllah Alaei, Mehrdad Shariati

**Affiliations:** 1 Department of Biology, Kazerun Branch, Islamic Azad University, Kazerun, Iran; 2 Department of Physiology, School of Medicine, Isfahan University of Medical Sciences, Isfahan, Iran

**Keywords:** Addiction, Central nucleus of amygdala, CPP, Deep brain stimulation, Dependence, Dopamine D2 receptor – antagonist, Morphine, Rat

## Abstract

**Objective(s):**

The central nucleus of the amygdala (CeA) is one of the most important areas for the morphine reward system. This study investigated the effect of electrical stimulation of CeA on morphine conditioned place preference (CPP) in male rats.

**Materials and Methods:**

After anesthetizing male Wistar rats, both electrode and cannula were implanted into CeA for stimulating (low intensity: 25 μA, and high intensity: 150 μA) and injecting (lidocaine and dopamine D2 receptor antagonist), respectively. Then, CPP induced by effective (5 mg/kg) and ineffective (0.5 mg/kg) doses of morphine was evaluated for five consecutive days (n = 6 / group).

**Results:**

The low electrical stimulation intensity of 25 μA suppressed both acquisition and expression phases, but the high intensity of 150 µA attenuated only the expression phase. On the other hand, intra-CeA administration of dopamine D2 receptor antagonist, eticlopride (2 µg/rat), with the effective dose of morphine, decreased CPP. In addition, infusion of lidocaine into the CeA inhibited morphine-induced CPP in both acquisition and expression phases with the effective dose of morphine.

**Conclusion:**

Electrical stimulation of the CeA may play an important role in attenuating morphine induced CPP via possible changes in neurotransmitters involved in the reward system such as dopamine (DA) and gamma-aminobutyric acid (GABA).

## Introduction

Morphine as an opioid drug is widely used in clinic practice for decreasing sever pain. On the other hand, there is a seriuos risk of addiction when taking this drug (1). Also, morphine abuse can lead to pain tolerance, hyperalgesia, physical dependence, and other negative effects (2). Morphine addiction induces the reward circuit to become overactive, leading to compulsive substance-seeking (3).

For decreasing morphine-induced addiction, several treatment methods have been suggested, despite high recurrence rates (4). Currently, deep brain stimulation (DBS) is known as one of the neurosurgical procedures (5)*.* DBS has been used to treat Parkinson’s disease (6), depression (7), Tourette syndrome (8), and Obsessive-Compulsive Disorder (OCD) (9). In this method, electrodes are inserted in some specific areas of the brain to induce different electrical pulses into the target sites (5). Additionally, some exprimental studies have reported the effects of DBS on treatment of drug addiction, especially in decreasing drug-seeking behavior (10-12). Also, some researchers demonstrated that DBS reduced addictive behavior in the brain nuclei such as nucleus accumbens (Nac) (12), lateral hypothalamus, lateral habenula (11), subthalamic nucleus (STN), and the insula (13, 14). 

The amygdaloid complex, especially the central nucleus of the amygdala (CeA) is one of the brain regions associated with drug reward that contain high concentrations of opioid receptors (15). CeA receives abundant DAergic afferents from the ventral tegmental region projected physically to the Nac and ventral tegmental area (VTA) (16). It also contains a higher number of DA terminals than other parts of amygdaloid nucleus. In addition, the CeA as the major output nucleus of the amygdala, plays a significant role in stimulus-reward learning (16-18). 

Conditioned place preference (CPP), the most common behavioral model, is frequently used to measure learning and memory in animal models (19). The underlying feature of CPP includes dependence of a certain environment on the medication treatment, followed by association of another environment with the absence of the drug (20). 

Previous studies have shown that morphine induces conditioned preference in rats (21, 22). Also, several experimental studies have revealed that the DBS of brain nuclei involved in the reward system reduces morphine-induced CPP (10, 23). In spite important role of CeA in reward system, the effect of electrical stimulation of CeA on morphine-induced CPP was unclear; therefore, this study was planned to investigate the effect of CeA electrical stimulation with high and low intensities on CPP induced by effective and ineffective doses of morphine in male rats. Also, the effects of reversible inactivation by lidocaine injection and antagonist of dopamine D2 receptor, ethiclopride, on CeA in CPP were evaluated.

## Materials and Methods

***Experimental design***

**Figure 1:**
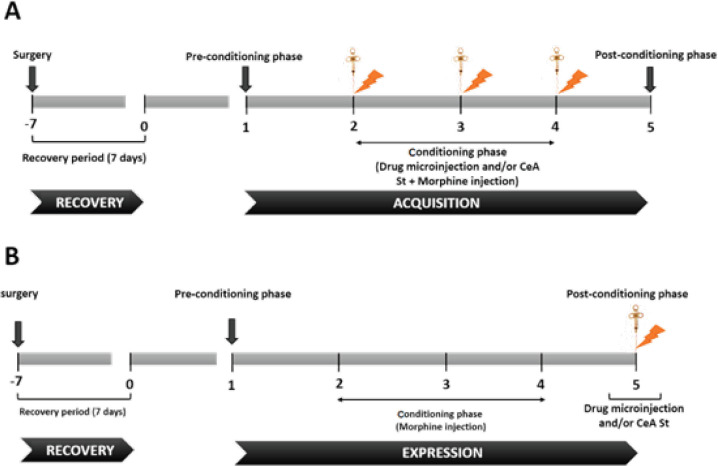
A: Schematic diagram of the experimental protocol for method of electrical stimulation or drug administration in the acquisition phase. B: Method of electrical stimulation or drug administration in the expression phase. Electrical stimulation: drug microinjection and morphine injection

In this experiment, animals were randomly divided into the following surgical groups (n = 6): saline (Sal), saline + electrical stimulation 25 (Sal + St 25), morphine 0.5 (Mor 0.5), morphine 0.5 + electrical stimulation 25 (Mor 0.5 + St 25), morphine 5 (Mor 5), morphine 5 + electrical stimulation 25 (Mor 5 + St 25), saline + electrical stimulation 150 (Sal + St 150), morphine 0.5 + electrical stimulation 150 (Mor 0.5 + St 150), subcutaneous saline (Sal SC), intracerebral saline (sal i.c), morphine 0.5 + lidocaine (Mor 0.5 + Lido), morphine 5 + lidocaine (Mor 5 + Lido), experession lidocaein (Exp Lido), and morphine 5 + Eticlopride (Mor 5 + Eticlopride). All above mentioned groups were assigned in both expression and aqusiation phases. According to previous studies, the morphine doses were chosen as effective (5 mg/kg) and ineffective (0.5 mg/kg) doses (22, 24). A shematic diagram of experimental design was illustrated in Figure 1.

***Animals***

This study was performed on male Wistar rats weighing 250–300 g obtained from Isfahan University, Isfahan, Iran. The animals were carried to the animal house to adapt for ten days before surgery. They were maintained under controlled conditions (12 hr light - 12 hr dark and temperature of 22 ± 2 °C) with available food and water. All animal experiments were approved by the Ethics Committee of Kazeroon Azad University (IR.IAU.KAU.REC.1400.061). Also, the experiments were done according to The National Institute of Health Guide for the Care and Use of Laboratory Animals (NIH Publication, 8th edition, 2011).

***Drugs***

The drugs used in the experiment were ketamine (100 mg/kg; TRITTAU Co, Germany), xylazine (10 mg/kg; Interchemie Co., Holland), Gentamicin (6 mg / kg; Alborz Darou Co Iran), Lidocaine 2% hydrochloride (0.3 μl per site ; Caspian Tamin Pharmaceutical Co. Iran), morphine (0.5 and 5 mg/kg; Pade Co., IRAN), and Eticlopride hydrochloride (2 µg/kg; Sigma-Aldrich Co, Germany). During the experiment, morphine and saline were subcutaneously (SC) administered as well as lidocaine and dopamine D2 receptor antagonist which were injected by intracerebral injection (i.c).

***Surgery***

The rats were anesthetized with ketamine (100 mg/kg) and xylazine (10 mg/kg). After shaving, the animal head was fixed in a stereotaxic apparatus (RWD Life science Co, China). Then, an incision along the midline was made to expose the skull. For observing the bregma and lambda areas, the skull surface was entirely cleaned. Next, both unilateral cannula and electrode were implanted into the CeA for stimulating and injecting, respectively. The coordinates of CeA included Anterior-Posterior (AP) = -2.2 mm; Medial-Lateral (ML) = ± 4.2 mm and Dorsal-Ventra (DV) = -8.4 mm (25). Afterward, both cannula and the electrode were fixed to the skull surface with dental acrylic cement (Acropars Co. Tehran Iran). To prevent infection, the animals received gentamicin (6 mg/kg; SC). Finally, they were transferred to Plexiglas cages to recover for 5 to 7 days.

***Behavioral procedure***

*CPP apparatus*

The place conditioning apparatus consisted of three metal chambers (A, B, and C). Both chambers A and B were equal-size (30 cm × 30 cm × 40 cm) separated by a guillotine door. The A chamber had black and white walls as well as a rough floor, while the B chamber had white walls and a smooth floor. Chamber C was the smallest (30 cm ×10 cm × 40 cm) linked to chambers A and B by a guillotine door. When the guillotine door was removed, the animal could freely move between the two chambers A and B through chamber C. CPP was performed on five continuous days using a biased procedure that included three phases of pre-conditioning, conditioning, and post-conditioning (26).

*Pre-conditioning phase*

During this phase (day 1), each rat was placed in chamber C to explore freely the three chambers for 15 min, while all doors of the apparatus had been removed. In addition, a camera placed above the apparatus recorded the time spent by each animal in chambers A and B. Whenever the animal spent 60% of its stopping time in a chamber, the opposite side was considered the morphine injection chamber.

*Conditioning phase*

The conditioning phase included days 2–4 of the study. In this phase, all groups received morphine and saline once per day. The phase contained six 30-min sessions (three saline and three morphine administrations). In addition, lidocaine, ethichlopride, and electrical stimulations were accompanied with morphine injection. On the 2nd day morning of the conditioning phase, the rat receiving morphine was immediately placed in the less-preferred chamber of the CPP apparatus. During the conditioning phase, all guillotine doors were closed for 30 min. After six hours, the animal received saline (1 ml/kg) and was immediately placed in the other chamber of the CPP apparatus, similar to the previous session. On the 3rd day, morphine and saline injections were the opposite of the 2nd day. Also, the morphine and saline injections on days 4 and 2 were the same. Moreover, the control group received saline twice per day.

*Post-conditioning phase*

The post-conditioning phase included the fifth day of the experiment. Each rat had a free choice in the three chambers of apparatus for 15 min while the guillotine doors had been removed. The time spent in the drug-paired chamber was recorded by ANY-Maze software (Stoelting Co, USA) and compared with the pre-conditioning phase. The changes of preference (preference index) were computed as the difference between the times spent in the morphine-paired chambers in post-conditioning and pre-conditioning phases (Preference index = time spend _post conditioning_ – time spend _precondithining_). 

***Electrical stimulation induction***

In this study, electrical intensities 25 or 150 μA were induced by a Stimulator Isolator A36O, (World Precision Instruments, CO, USA). The electrical intensities were chosen according to the protocol used for stimulation of the prelimbic cortex of medial prefrontal cortex (mPFC) (12). Ten min before administration of morphine, each animal was stimulated by electrical intensities of 25 or 150 μA with a constant frequency of 25 Hz, once every five seconds for 10 min. In addition, the acquisition and expression groups received electrical stimulation during the conditioning and post-conditioning phases, respectively.

***Histology***

**Figure 2:**
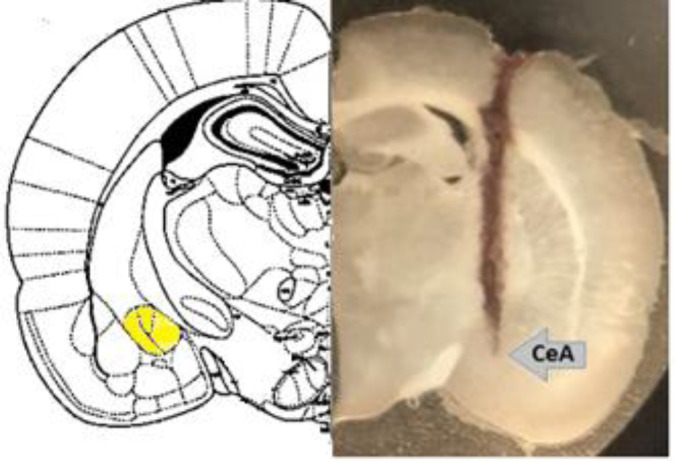
Location of electrode or cannula in the central nucleus of the amygdala (CeA) of rat brain. Arrow points to the CeA location (magnification 400 x)

In order to confirm the stimulating electrode or cannula site in the CeA, a histological evaluation was performed. At the end of the experiment, the rats were anesthetized with ketamine and xylazine. Then, they were transcardially perfused with a saline solution of 0.9% followed by a formalin solution of 10%. The brain tissues were removed and kept in the formalin solution for one week. Then, the tissue slices with a thickness of 60 μm were prepared by frize-microtome. The sections were examined by an optical microscope (ERMA, Tokyo, Japan) and compared with the rat brain atlas (Figure 2)(12). 

***Statistical analysis***

All data were expressed as mean ± standard error of the mean. The data were analyzed using one-way analysis of variance (ANOVA) followed by Tukey *post hoc* test by SPSS software version 16. The statistical differences with *P*<0.05 were considered to be significant. 

## Results

***Effect of CeA electrical stimulation with various doses of morphine on the acquisition phase of CPP***

**Figure 3:**
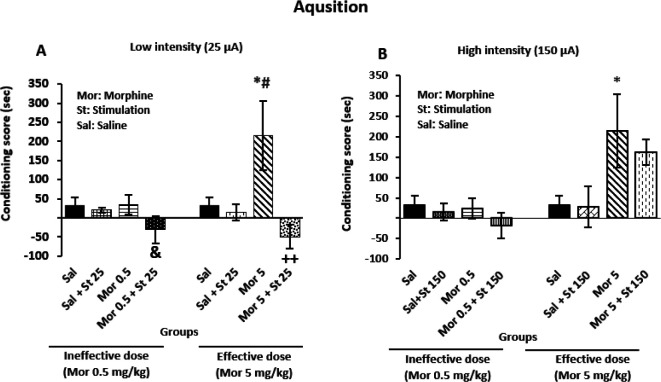
Effect of electrical stimulation with low and high current intensities on CeA in the acquisition phase of conditioned place preference (CPP)in male rats. One-way analysis of variance followed by Tukey *post hoc *test showed that A) Electrical stimulation of CeA with the low intensity in combination with both effective and ineffective doses of morphine significantly suppressed CPP in the acquisition phase. B) Unilateral electrical stimulation of CeA with high current intensity with both effective and ineffective doses of morphine had no significant effect on the acquisition phase of CPP. *P<0.05 compared with the sal group; #*P*<0.05 compared with the sal +St 25 group; ++*P*<0.01 compared with the Mor 5 group; &*P*<0.05 compared with the Mor 0.5 group

In this study, the low current intensity in combination with effective (*P*=0.006) and ineffective (*P*=0.03) doses of morphine reduced the acquisition phase in the CPP when compared with the Mor group (Figure 3A). In contrast, high current intensity accompanied with effective (*P*=0.873) and ineffective (*P*=0.807) morphine doses did not significantly decrease the acquisition phase compared with morphine alone treated groups (Figure 3B). Also, the effective dose of morphine alone increased CPP compared with Sal (*P*=0.03) and Sal+ St 25 (*P*=0.03) groups, significantly (Figure 3A). The high current intensity in combination with effective (*P*=0.873) and ineffective (*P*=0.807) doses of morphine could not attenuate CPP in the acquisition phase of CPP in comparison with morphine alone treated groups, while the effective dose of morphine induced CPP in comparison with the sal group, significantly (*P*=0.045) (Figure 3B).

***Effect of CeA electrical stimulation with various doses of morphine on the expression phase of CPP***

**Figure 4:**
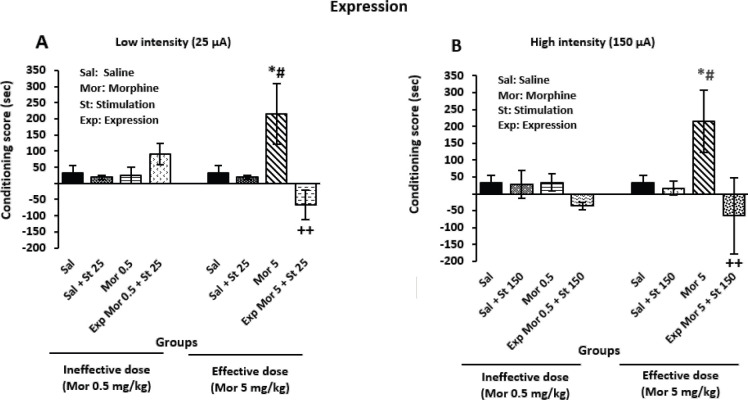
Effect of electrical stimulation with low and high current intensities on the central nucleus of the amygdala (CeA) in the expression phase of conditioned place preference (CPP) in male rats

The findings showed that administration of the morphine effective dose alone increased CPP in the expression phase in comparison with sal (*P*=0.035) and Sal + St 25 (*P*=0.044) groups, significantly (Figure 4A). Also, electrical stimulation of CeA with low intensity in combination with the effective dose of morphine significantly suppressed CPP in the expression phase in comparison with the Mor 5 group (*P*=0.003) (Figure 4A) Moreover, the effective dose of morphine alone induced a significant increment in CPP compared with Sal (*P*=0.05) and sal + St 150 (*P*=0.05) groups (Figure 4B). Also, CeA stimulation with high current intensity in combination with the effective dose of morphine could significantly suppress morphine-induced CPP compared with the Mor 5 group (*P*=0.006) (Figure 4B). On the other hand, the ineffective dose of morphine in combination with either low (*P*=0.112) or high (*P*=0.158) current intensity exhibited no significant effects (Figure 4A, B).

***Effect D2 receptor antagonist microinjection into CeA on the acquisition phase of morphine-induced CPP***

**Figure 5:**
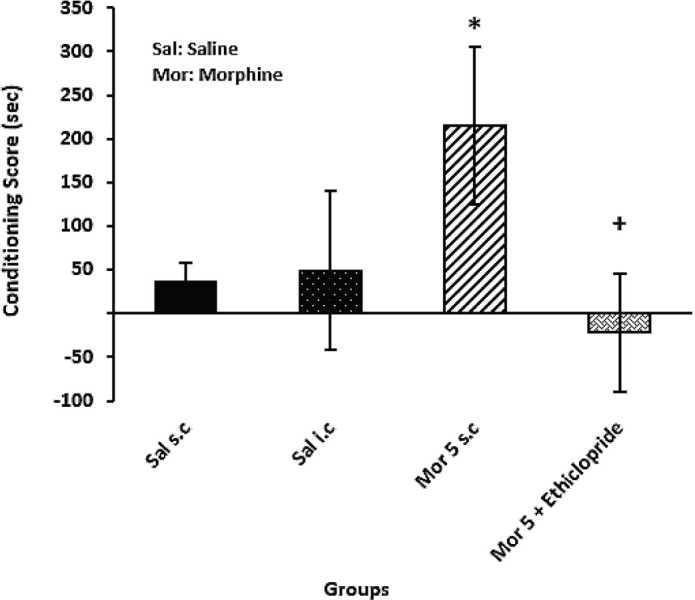
Effect of eticlopride on the acquisition phase of conditioned place preference (CPP) in male rats. One-way analysis of variance followed by Tukey *post hoc* test showed that eticlopride in combination with the effective dose of morphine significantly inhibited the acquisition phase of CPP compared with mor 5 SC group. +*P*<0.05 compared with the Mor group; *P<0.05 compared with the Sal SC group

The results showed that administration of eticlopride into CeA with the effective dose of morphine could significantly attenuate conditioning score in comparison with Mor 5 SC group (*P*=0.03). In contrast, administration of Mor 5 enhanced conditioning score compared with the Sal SC group (*P*=0.05) (Figure 5).

***Effect of lidocaine reversible inactivation on the CeA***

**Figure 6:**
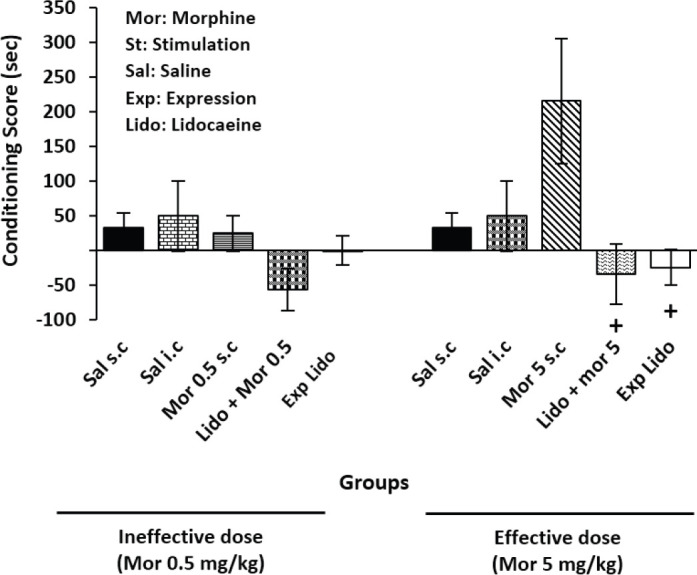
Effect of lidocaein reversible inactivation on the central nucleus of the amygdala (CeA) in both acquisition and expression phases of conditioned place preference (CPP) in male rats. One-way analysis of variance followed by Tukey *post hoc* test showed that lidocaine in combination with effective dose of morphine significantly inhibited both expression and acquisition phases of CPP compared with Mor5 SC group, +*P*<0.05 compared with the Mor 5 SC group

Injection of lidocaine into the CeA in combination with the effective dose of morphine in both acquisition (*P*=0.*02*) and expression *(P*=0.*03*) phases reduced morphine-induced CPP in comparison with the Mor 5 SC group, significantly. Also, the observation was detected in combination with the ineffective dose of morphine, but was not significant (*P*=0.48) (Figure 6).

## Discussion

The purpose of this study was to evaluate the effect of electrical stimulation of CeA on morphine-induced CPP in male rats. 

The current study results showed that CeA electrical stimulation with current intensity of 25 µA in combination with the ineffective dose of morphine could suppress the acquisition phase of morphine-induced CPP compared with the Mor group (Figure 3A). In contrast, stimulation of other areas of the brain such as the prelimbic cortex of mPFC enhanced the morphine-induced CPP in the rat (12). Several investigations on mPFC and NAc indicated that electrical stimulation in combination with the ineffective dose of morphine induced CPP (24, 27). However, some researchers reported different effects of electrical stimulation on CPP (26, 28). It is important to notice that CPP is a learning model that requires formation of associations between reward and particular location (29). Since the CeA has a role in morphine-dependent memory retrieval (30), absence of morphine-induced CPP in this study may be attributed to a decrease in the reward signal or an insufficient response to rewarding stimuli, which would impair learning and memory formation throughout the conditioning phase (Figure 3B).

Moreover, the present investigation showed that CeA stimulation with current intensity of 150 µA in combination with 5 mg/kg dose of morphine could block CPP in the expression phase; although, the low intensity blocked CPP in both phases (Figure 4 B). In agreement with these findings, previous research demonstrated that peripheral electrical stimulation inhibited both the expression of morphine-induced CPP and reactivation of extinguished CPP (27). 

In the present study, it is possible that electrical stimulation of CeA induced the release of gamma-aminobutyric acid (GABA) in CeA. The increment of GABA reduces DA release in VTA (31) resulted in decreasing emotional state and memory conditioning via the DAergic afferents originating from VTA (32). The GABA neurons are the dominant cells in the CeA projected to the VTA neurons. They regulate the activity of DAergic neurons in the VTA implicated in opioid reward (33-35). As a result, the blocking and activating of CeA GABA receptors may affect opioid reward behavior (31, 36). 

The present study showed that microinjection of dopamine D2 receptor antagonist, eticlopride, into the CeA with the effective dose of morphine decreased morphine conditioning (Figure 4). Rezayof *et al.* reported that dopamine D2 receptor antagonist decreased morphine-induced CPP dose-dependently. Also, the drug attenuated the potentiation induced by dopamine D2 receptor agonist (37). Thus, inhibition of DA D2 receptors by eticlopride probably blocked the reward-related motivation learning. In line with this possibility, some studies proposed that dopamine receptor agonists could support reward-related motivation learning, while antagonists prevent the typical effects of reward on behavior (38, 39). An evidence also presented NMDA receptor antagonist, impairing learning and memory, could prevent opiate dependence (40). Furthermore, some studies showed that the amygdala may play a critical role in stimulus-reward learning (18, 41). The CeA lesion before conditioning impaired the acquisition of the conditioned responses (42). Therefore, either activation or inhibition of dopamine D2 receptors may influence the CeA-associated memory. In this study, the effect of electrical stimulation on CPP was similar to the dopamine antagonist. The object reveals that electrical stimulation could be an appropriate way to understand and detect the areas and mechanisms involved in addiction (Figure 5). 

In addition, the present study decided to investigate the effect of CeA inactivation by lidocaine on morphine-induced CPP. Lidocaine hydrochloride is a transitory inhibitor of steady-state tetrodotoxin-sensitive sodium channels. It reversibly blocks neuronal action, unlike lesion. Lidocaine neuronal inactivation allows study of the role of particular brain areas in learning and memory (43). In accordance with this, the current findings showed that injection of lidocaine into the CeA accompanied with morphine effective dose significantly reduced morphine-induced CPP during both expression and acquisition phases (Figure 6). As mentioned above, CeA has a key role in reward-related memory, therefore, morphine-dependent learning is probably linked to the rewarding effects of morphine (18) proven by the present study.

## Conclusion

The present study showed that electrical stimulation of CeA with low current intensity in combination with both morphine effective and ineffective doses blocked morphine-induced CPP in both expression and acquisition phases. It is possible that electrical stimulation of CeA disrupted memory and learning in the paradigm by changing concentration of DA resulting from changing the GABA concentration. The possibility was approved by the effects of electrical stimulation and DA antagonist on CPP. Conversely, inactivation of CeA by lidocaine disrupted the morphine-induced CPP indicating the important role of CeA in learning.

## Authors’ Contributions

HAA Conceived the study and design; ZJ and HAA Analyzed data and prepared the draft manuscript; ZJ, HA A, SK, and MS Critically revised the paper; ZJ, HA, SK, and MS Supervised the research; ZJ, HA A, SK, and MS Approved the final version to be published.

## Funding

This research was financially supported by the Kazerun Branch, Islamic Azad University, Kazerun, Iran (IR.IAU.KAU.REC.1400.061). We appreciate all who assisted us in this research.

## Conflicts of Interest

The authors declare that they do not have any conflicts of interest.
